# Effect of Resin Cement at Different Thicknesses on the Fatigue Shear Bond Strength to Leucite Ceramic

**DOI:** 10.1055/s-0042-1758797

**Published:** 2022-12-19

**Authors:** Laura Viviana Calvache Arcila, Laís Carolina Landim Gomes, Laura Patricia Nadal Ortiz, Mardoqueu M. da Costa, João Paulo Mendes Tribst, Marco Antonio Bottino, Guilherme de Siqueira Ferreira Anzaloni Saavedra, Renata Marques de Melo

**Affiliations:** 1 Department of Dental Materials and Prosthodontics, Institute of Science and Technology, São Paulo State University (Unesp), São José dos Campos, Brazil; 2 Department of Dentistry, Brasil University, São Paulo, Brazil; 3 Department of Oral Regenerative Medicine, Academic Centre for Dentistry Amsterdam (ACTA), University of Amsterdam and Vrije Universiteit Amsterdam, Amsterdam, The Netherlands

**Keywords:** ceramic, cement, failure load, fatigue, shear strength

## Abstract

**Objectives** This *in vitro* study was performed to evaluate fatigue survival by shear test in the union of leucite-reinforced feldspathic ceramic using different cement thicknesses.

**Materials and Methods** Leucite-reinforced glass ceramics blocks were sectioned in 2-mm thick slices where resin cylinders were cemented. The samples were distributed in two experimental groups ( *n* = 20) according to the cement thickness (60 and 300 μm). The specimens of each group were submitted to the stepwise fatigue test in the mechanical cycling machine under shear stress state, with a frequency of 2 Hz, a step-size of 0.16 bar, starting with a load of 31 N (1.0 bar) and a lifetime of 20,000 cycles at each load step.

**Results** The samples were analyzed in a stereomicroscope and scanning electron microscopy to determine the failure type. There is no significant difference between the mean values of shear bond strength according to both groups. Log-rank ( *p* = 0.925) and Wilcoxon ( *p* = 0.520) tests revealed a similar survival probability in both cement layer thicknesses according to the confidence interval (95%). The fracture analysis showed that the mixed failure was the most common failure type in the 300-μm thickness group (80%), while adhesive failure was predominant in the 60-μm thickness group (67%). The different cement thicknesses did not influence the leucite ceramic bonding in fatigue shear testing; however, the thicker cement layer increased the predominance of the ceramic material failure.

**Conclusion** The resin cement thicknesses bonded to leucite ceramic did not influence the long-term interfacial shear bond strength, although thicker cement layer increased the ceramic material cohesive failure. Regardless the cement layer thickness, the shear bond strength lifetime decreases under fatigue.

## Introduction

Dental ceramics are materials routinely used in aesthetic restorative procedures due to their excellent properties, such as high compressive strength, high translucency and fluorescence, chemical stability, low electrical conductivity, and the similar thermal expansion coefficient to the dental substrate. 1

The cementation of dental ceramics is a widely studied clinical step, which should be done meticulously to achieve a durable bond strength to the dental substrate. 2 3 Thus, resin cements are the first choice materials during the cementation of glass-ceramics restorations, as they provide the advantage of mechanical bonding in addition to the chemical adhesion provided by silanes coupling agent. 4 5

Several factors affect the bond strength of the resin cement with the restoration, such as the restoration material, the adhesive system, the cement polymerization mode, the different surface treatments, the proper application of silane coupling agent, and the presence of surface contaminants. 3 4 5 6

In addition to the reported parameters, literature shows the influence of resin cement thickness on the fracture resistance of ceramic restorations. 7 The authors analyzed the influence of cement thickness and ceramic/cement bonding on the stresses and failures of computer-aided design/computer-aided manufacturing (CAD/CAM) crowns using finite element analysis and monotonic tests. The major findings were that the occlusal “fitting” may promote structural implications in the CAD/CAM crowns; and precementation spaces around 50 to 100μm are recommended. 7

According to the literature, the benefits of adhesive dentistry are dampened with a cement thickness close to 450 to 500μm due to polymerization shrinkage stresses. 6 7 However, a previous report concluded that the cement layer thickness did not interfere in the mechanical performance of ceramic restorations. 8 9 However, there is a lack of information in literature regarding the influence of cement thickness on the bond strength durability.

Besides to the previously described, the presence of moisture and chewing loads in the oral cavity are factors capable of impairing the restoration longevity with the degradation of cement layer, slow crack growth, and fatigue. 10 The masticatory loads promote mechanical degradation, whereas moisture corrodes the chemical bonds at the ceramic crack tip. In the same manner, moisture from saliva and dentinal tubule can also greatly influence the degradation of resin cements, and finally leads to failure at the adhesive interface. 10 However, the survival probabilities regarding the fatigue of ceramic bonded to cements have not been assessed so far.

Therefore, there is lack of scientific evidence in literature showing the influence of mechanical fatigue on the resin cement and glass ceramic bond strength survival. The objective of this study was to assess the bond strength survival of leucite-reinforced feldspathic ceramic (IPS Empress CAD Multi, Ivoclar Vivadent, Schaan, Liechtenstein) during shear stress using different cement thicknesses. The null hypothesis was that the different cement thicknesses will not influence the fatigue shear bond strength of leucite-reinforced feldspathic ceramic.

## Materials and Methods

First, specimens were made using leucite-reinforced glass ceramics to evaluate the influence of cement thickness on the shear bond to leucite-reinforced feldspathic ceramics, where a resin cylinder was cemented on one of the sides for it to be subjected to the mechanical shear test. The materials used in this study are shown in Table 1 .

**Table 1:** Trade market, batch number, manufacturer, and chemical composition of the materials used in this in vitro study

Product	Material type	Chemical composition	Batch number	Manufacturer
Empress CAD	Leucite-reinforced glass ceramic	SiO _2_ : 60.0–65.0 Al _2_ O _3_ : 16.0–20.0 K _2_ O: 10.0–14.0 Na _2_ O: 3.5–6.5 Other oxides: 0.5–7.0 Pigments: 0.2–1.0	X49765	Ivoclar Vivadent AG, Liechtenstein
Multilink N	Resin cement	Alcohol solution of silane methacrylate, phosphoric acid methacrylate, and sulfide methacrylate	W44613	Ivoclar Vivadent AG, Liechtenstein
Condac Porcelana (5%)	Hydrofluoric acid	10% hydrofluoric acid, water, thickener, surfactant, and pigment	030518	FGM; Joinville, SC, Brazil
Opallis	Microhybrid composite resin	Bis-GMA monomers, Bis-EMA, TEGDMA, UDMA, camphorquinone, co-initiator, silane, silanized barium-aluminum silicate glass, pigments	240516	FGM; Joinville, SC, Brazil
Monobond N	Silane	3-Methacryloxypropyltrimethoxysilane, ethanol, water	W90329	Ivoclar Vivadent AG, Liechtenstein

Abbreviations: Bis-EMA, bisphenol A ethoxylated dimethacrylate; Bis-GMA, bisphenol A-glycidyl methacrylate; TEGDMA, triethylene glycol dimethacrylate; UDMA, urethane dimethacrylate.

### Ceramic Processing

Ceramic blocks (IPS Empress CAD Multi) were obtained and then sectioned in a standardized manner using a precision cutter (Isomet 1000; Buehler, Lake Bluff, Illinois, United States) under constant water cooling to obtain 2-mm thick slices (5 × 5 mm). A total of 40 slices were obtained, which were polished in a polishing machine with 600 grit sandpaper under constant water cooling. Then, they were randomly distributed into two experimental groups according to the cement thickness used in the cementation of the ceramic ( *n* = 20).

### Resin Cylinders Manufacturing

Composite resin cylinders (Opallis, FGM, Joinville, Brazil) (3.2 mm × 4 mm) were made using a standardized silicone matrix and the incremental technique under a glass slide to obtain a completely smooth surface, and light curing was performed for 30 seconds on each layer with light source unity (Blue Phase, Ivoclar Vivadent) at a light intensity of 1,200 mW/cm ^2^ .

### Standardization of Cement Thickness

**Fig. 1:**
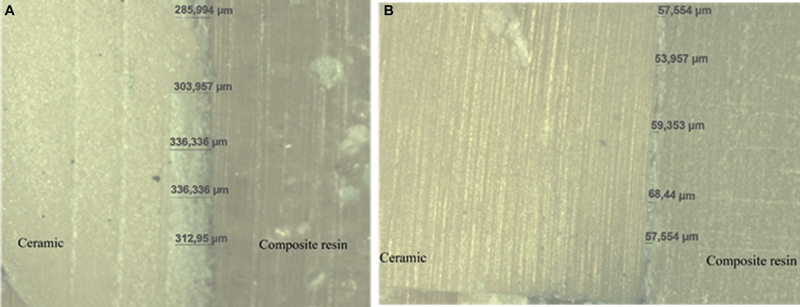
Stereomicroscope analyses to standardize the thickness cement of the groups under magnification of 24.5 × . ( **A** ) 300 μm group, ( **B** ) 60 μm group.

To standardize the cement thickness, a pilot study was performed in which the specimens were cemented witch different weights at static load. After the cementation, the specimens were embedded in acrylic resin cylinders (2.5 cm × 2.5 cm) and cut into halves using a precision cutter (Isomet 1000; Buehler). Then, each sectioned sample was submitted to the microscopy analysis (Discovery V20, Carl Zeiss, Jena, Germany) aiming to define the average thickness generated by each load. In the end, the control group (60 μm thickness) was achieved with a load of 51.2 g and the second group (300 μm thickness) received a higher weight of 811 g ( Fig. 1 ). Therefore, in the present study one group presented a thin cement layer (60 μm) and the other group presented 5× this cement layer thickness (300 μm).

### Cementation of Specimen

**Fig. 2:**
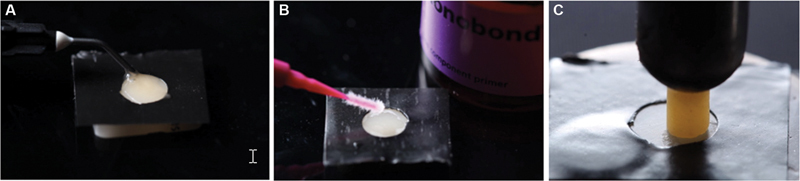
Cementation process with a perforated matrix. ( **A** ) Isolated surface etching with hydrofluoric acid 5%. ( **B** ) Silane applying with the aid of a microbrush. ( **C** ) Cementation of the resin cylinders using static loading.

**Fig. 3:**
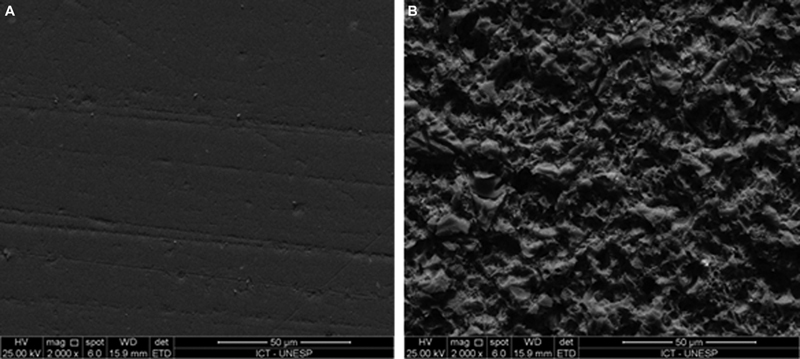
Topography micrographs of ceramic under 2000× magnification. ( **A** ) Polished ceramic. ( **B** ) Acid etched ceramic.

Next, the ceramic surface was isolated with a perforated matrix correspondent to the adhesive area of the cylinders samples to avoid excess of cement ( Fig. 2 ). The isolated surface was etched with hydrofluoric acid 5% ( Fig. 2A ) (Condac Porcelain, FGM) for 60 seconds, washed with water for 60 seconds, and then a jet of air was used for 15 seconds to dry the surface. The silane (Monobond N, Ivoclar Vivadent) was applied with the aid of a microbrush for 60 seconds ( Fig. 2B ). After surface treatments, the resin cement (Multilink N, Ivoclar Vivadent) pastes were mixed as recommended by the manufacturer and applied on the center of the treated ceramic surface and on its counterpart on the resin cylinders. The ceramic and the cylinders were bonded together and a static load of 51.2 g weight was applied to the top surface of the cylinder to the 300-μm group; and a load of 810 g was used to the 60-μm group ( Fig. 2C ). Then, the cement excesses were removed, and light activation (1200 mW/cm ^2^ , Bluephase N, Ivoclar Vivadent) was performed in five exposures of 20 seconds on each side of the sample. The etched ceramic surface and the polished ceramic can be observed with scanning electron microscope (SEM) in Fig. 3 .

### Monotonic Test

**Fig. 4:**
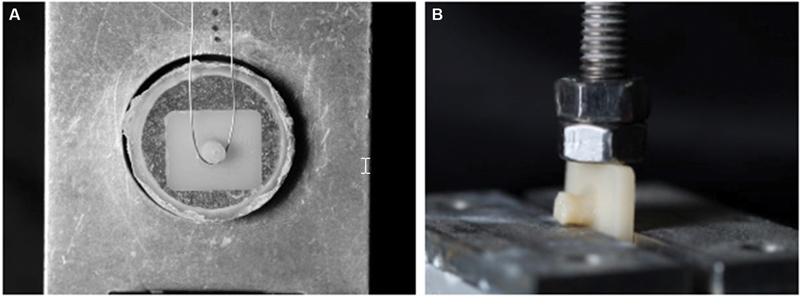
( **A** ) Monotonic test and ( **B** ) fatigue test device.

The immediate bond strength test was performed to determine the fatigue survival test profiles. To do so, a microshear assay (DL-1000, EMIC, Instron, São Jose dos Campos, Brazil) was performed with a load cell of 20 kgf (0.5 mm/min) in the samples ( *n* = 6) of both groups ( Fig. 4A ).

### Stepwise Fatigue Test

The stepwise test corresponds to a simulation of a clinical situation through the cycling of the samples with increasing load until material failure or up to a predetermined number of cycles. 11 12 13 14 The fatigue test parameters were defined according to the monotonic test results: the frequency was 2 Hz, with step size of 0.16 bar, starting with a load of 31 N (1.0 bar) during 20,000 cycles at each load step until the survival or failure of the sample (suspension). The specimens of each group ( *n* = 20) were submitted to a fatigue test in a mechanical cycler (Biocycle, BIOPDI, São Carlos, São Paulo, Brazil). The uniaxial load was applied with a 12-mm diameter stainless steel piston in the lateral surface of the ceramic, which was supported and arrested between two flat steel bases perpendicular to the load incidence. The test was performed with the samples submerged in water ( Fig. 4B ).

### Failure Analysis

**Fig. 5:**
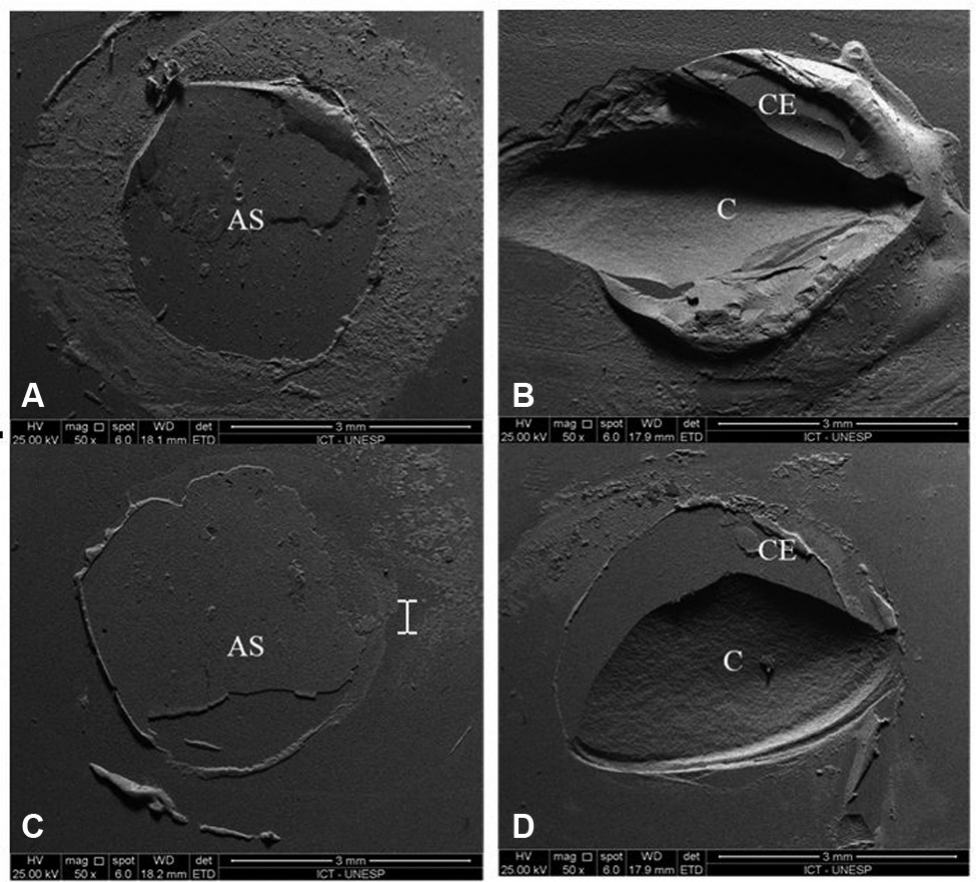
Failure type under 50 X magnification. ( **A** ) Adhesive failure in the 300-μm group. ( **B** ) Mixed ceramic failure in the 300-μm group. ( **C** ) Adhesive failure in the 60-μm group. ( **D** ) Mixed ceramic failure in the 60-μm group. C, ceramic; CE, cement; AS, adhesive surface.

After the fracture, the samples were analyzed in a stereomicroscope (Discovery V20, Carl Zeiss) under magnification of 7.5× to determine the failure type, and representative specimens ( *n* = 3) from each group have been analyzed with SEM (FEG-SEM, Inspect S50; FEI Company, Brno, Czech Republic) in high vacuum, using a voltage of 25 Kv. Previously, the samples were metallized using the EMITECH SC7620 Sputter Coater metallizer, resulting in a 12-nm gold alloy layer over each sample. Two distinct types of failure were recorded: (1) adhesive failure was recorded when the bond failure was observed in the ceramic and (2) mixed failure was recorded when involving the cement and ceramic surface. The images were observed in secondary electrons and backscattered electrons at low and high magnification ( Fig. 5 ).

### Data Analysis

The fatigue data were subjected to a Kaplan–Meier analysis with post hoc Mantel–Cox (log-rank) and Wilcoxon test ( *α* = 0.05) (IBM SPSS Software; IBM, Armonk, New York, United States), as well as a reliability analysis by the Weibull test (Weibull ++, Reliasoft, Tucson, Arizona, United States). The mean between monotonic and fatigue test was calculated. The failures were classified and the percentages of each type of failure computed.

## Results

**Fig. 6:**
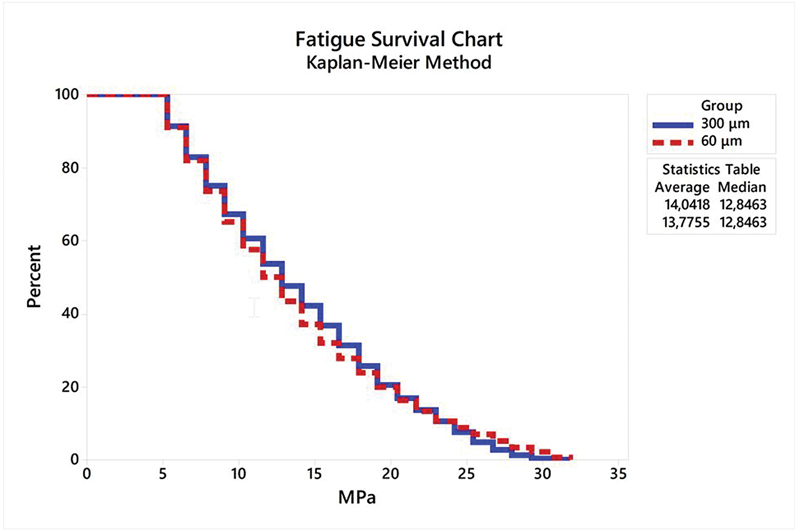
Survival plot using the Kaplan–Meier method, average and median strength of samples during the fatigue test.

**Fig. 7:**
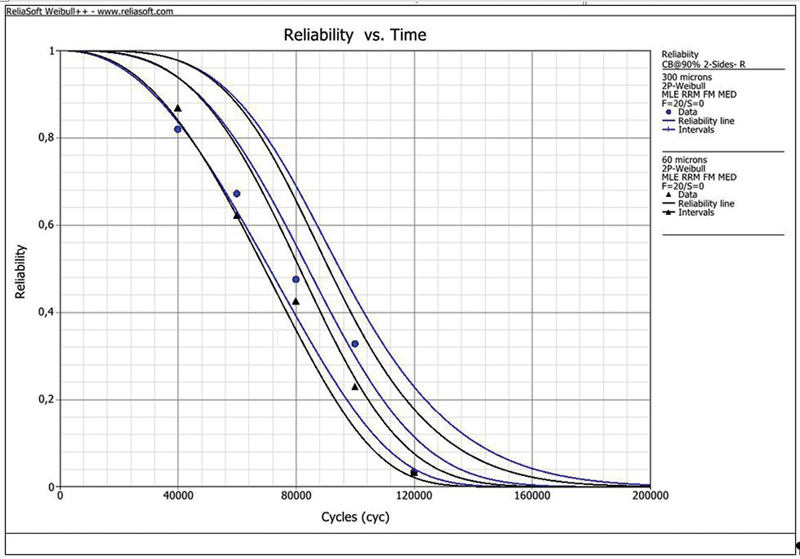
Reliability plot showing that samples will fail as the time under fatigue increases in both groups. The Weibull modulus ( *β* ) and characteristic life ( *η* ) for 300 and 60 µm groups were respectively: *β* = 3.2 and 3.3 and *η* = 94,000 and 90,000 cycles.

The Kaplan–Meier fatigue survival chart and the Weibull probability plots versus number of cycles during the fatigue test are presented in Figs. 6 and 7 , respectively. There is no significant difference between the mean values of shear bond strength according to both groups comparison. Log-rank ( *p* = 0.925) and Wilcoxon ( *p* = 0.520) tests revealed a similar survival probability in both cement thickness groups (300 and 60 μm) according to the confidence interval ( Table 2 ). In addition, the sample failure during the fatigue test increased progressively with the increase in the number of cycles and load (N). The Kaplan–Meier estimates are presented in Table 3 . No differences in mean values of bond strength (MPa) were observed between the two thicknesses for monotonic and fatigue tested interfaces ( Table 4 ).

**Table 2:** Survival probability in both cementation thickness groups (300 and 60 μm) according to the confidence interval of log-rank ( *p* 0.925) and Wilcoxon ( *p* 0.520) method

Method	Chi-squared	DF	*p* -Value
Log-rank	0.008764	1	0.925
Wilcoxon	0.413978	1	0.520

Note: Kaplan–Meier analysis with post hoc Mantel–Cox (log-rank) and Wilcoxon test ( *α* = 0.05). Abbreviation: DF, Degree of freedom.

**Table 3:** Results of shear bond fatigue test in MPa, number under risk, surviving specimens, survival probability, standard error, and confidence intervals (CIs)

Cement thickness	MPa	Number under risk	Surviving specimens	Survival probability	Standard error	CI of 95.0%	
						Lower	Upper
300 μm	5.28	236	20	0.915254	0.0181290	0.879722	0.950786
	10.32	159	16	0.605932	0.0318084	0.543589	0.668275
	15.36	100	13	0.368644	0.0314040	0.307093	0.430195
	20.40	49	9	0.169492	0.0244225	0.121624	0.217359
	25.44	18	6	0.050847	0.0143003	0.022819	0.078876
	30.47	1	1	0.000000	0.000000	0.000000	0.000000
60 μm	5.28	225	20	0.911111	0.0189722	0.873926	0.948296
	10.32	147	17	0.577778	0.0329276	0.513241	0.642315
	15.36	84	12	0.320000	0.0310984	0.259048	0.380952
	20.40	45	8	0.164444	0.0247119	0.116010	0.212879
	25.44	20	4	0.071111	0.0171340	0.037529	0.104693
	30.47	5	3	0.008889	0.0062574	0.000000	0.021153

Note: Kaplan–Meier analysis with post hoc Mantel–Cox (log-rank) and Wilcoxon test ( *α* = 0.05).

**Table 4:** Mean values of bond strength (MPa) obtained in monotonic and fatigue tests

Groups	Mean bond strength (MPa)	
Cement thickness	Monotonic test	Fatigue test
300 μm	23.32	20.52
60 μm	26.86	19.96

**Fig. 8:**
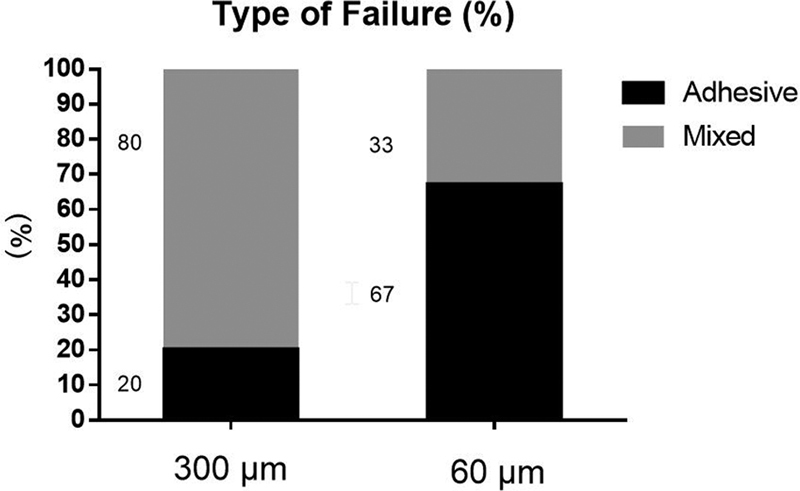
Type of failure data of each group (%) regarding the scanning electron microscope (SEM) analysis.

The fracture analysis showed that predominantly ceramic mixed failure was the most common failure type in the 300-μm thickness group (80%), followed by adhesive failure (20%). Moreover, the adhesive failure was predominant in the 60-μm thickness group (67%), followed by mixed failure (33%). The results (%) are shown in Fig. 8 . The images of failed specimens of each group and the topography micrographs polished and etched of ceramic obtained with the SEM are shown in Figs. 4 and 5 , respectively.

## Discussion

The goal was to assess the bond strength survival of leucite-reinforced feldspathic ceramic during shear stress using different cement thicknesses. The results showed that the cement thickness did not influence the bond strength survival, thus confirming the null hypothesis.

The leucite-based ceramic (SiO2, Al2O3, K2O, Na2O, other oxides, and pigments) are rich in silica, being considered an acid-sensitive ceramic. 4 It is in the glass-matrix structure that the hydrofluoric acid will modify the surface topography and form micro-retentions which improve the bond strength with cement material. Thus, this type of dental ceramic presents a strong bond strength with resinous cements, which endorses our findings. However, the literature reports that the polymerization shrinkage during light curing generates residual stress at the adhesive interface and reduces the adhesion durability of bonded ceramics with a thick cement layers. 15 16 Overall, the matter regarding cement thickness has been much explored in fiber post-studies; and a consensus about the association of thinner cement layer and higher bond strengths already exists. 17 18 Thus, according to the previous studies, the presence of voids and bubbles are responsible for the decrease of bond strength in thicker cement layers.

However, the well-controlled scenario during cementation and the flat surfaces in the present study probably impaired the inclusion of such defects and no differences were found between 300 and 60 μm cementation layers.

In addition to the defects population on the cement, Fleming et al 19 found that the volumetric shrinkage of the resin cement generates compressive stress at the interface, producing residual stress that allow the crack growth phenomena. 19 On the other hand, marginal discrepancy and lower bond strength caused by thicker resinous materials could be more evident in complex geometry, such as crowns, due to high polymerization shrinkage of the cement. 20 21 In addition, a thicker cement film exposes more polymeric material to the oral environment, increasing its susceptibility to the degradation. 22 23 24 It was reported that the acceptable cement thickness for dental restorations should be up to 120 μm. 25 Therefore, in the present study one group presented a clinically acceptable thickness (60 μm) and the other present 5× the cement layer height (300 μm). Despite this difference, the bond strength was similar between both.

One important aspect in bond strength studies is the failure mode evaluation. Adhesive failures occurs when there is no critical damage caused in the ceramic surface, and this factor is particularly important because these results are closer to the real bond strength between ceramic and cement. On the other hand, the thicker cement layer (300 μm) presented predominantly failures involving the ceramic substrate, with chipping of the ceramic surface. This result was caused probably due to the increase in the momentum during the test and the residual stress by the bigger cement volume. This may clinically signify that a cushioning effect between the crown and dentine due to the presence of a low elastic modulus layer (cement) under a much stiffer material (ceramic crown) is potentially lost in the 300-μm cement layer, thus leading to tensile fracture of the ceramic at the surface. 24 25

The reliability of both groups was the same and express that the bond strength will diminish over time due to fatigue, and 63.2% of the specimens will fail by around 90,000 cycles. In this study, a mechanical fatigue machine was applied for the aging process, but the specimens were immerged in water which can also contribute to cement plasticization and interfacial degradation.

For the lifetime prediction of a fatigue test to be considered clinically relevant, the evaluated restorations should be evaluated for at least 10 ^6^ cycles. This period of aging would correspond to approximately 1 year of clinical use. Considering three periods of 15 minutes of chewing per day, the individual average of chewing is 2,700 times a day with a frequency of 1 Hz. 26 These parameters may vary depending on the applied load and frequency.

The results obtained in our study regarding the survival analyses (Kaplan–Meier) show that more than half of the samples in both groups (300 and 60 μm) failed with approximately 20.40 MPa, and consequently the survival probability drops by a half with every cycle. The reliability plot also showed the same trend, as reliability decreased over time under fatigue. This shows the necessity of further analysis using fatigue tests to predict the long-term behavior of bonded restorations.

Dal Piva et al 27 observed shear stress and tensile stress simultaneously; however, they found high shear stress at the interface of the tested materials than tensile stress. In this study, the shear bond test was performed for monotonic and fatigue tests, in which the monotonic values were used to obtain the fatigue profile and load steps for the survival curve of each thickness group. This methodology was widely applied in studies that evaluated failures in restorative materials, 12 14 28 29 however, never with the present setup to test shear bond durability.

In this study, the cement thickness was standardized by applying a specific amount of weight using an adapted apparatus device for each specimen ( Fig. 2A ). The study of Ustun and Ayaz 30 used digital pressure when the resin cement was applied to the ceramic surfaces and seated to the dentin, which technically is not a standardizable procedure since there is no measured and control, and different pressures could be applied depending of the operator. A previous study applied different weights using a standardized pressure device, 31 and similar to the present study the authors were able to control the cement layer thickness; however, they also reported the need to check each sample under microscopy prior to the bond strength test due to the technique sensitivity. 31

According to the results of this study, the different cement thicknesses in the adhesive interface with leucite-reinforced feldspathic did not influence the fatigue behavior during cyclic shear stress state. However, we still advocate the control of cement thickness for better results in ceramic crowns restorations. 32

An important limitation inherent from the present study was the load application method. When testing adhesion using shear bond test, a combination of shear and tensile forces occur at the interface, resulting in complex stresses. In addition, the jig design applied in the *in vitro* setup show variations complicating direct comparison between studies. 33 Therefore, the comparisons between different shear bond strength methods should be carefully performed since the stress distributions have nonuniform tensile or shear stress states at the interface due to different specimens' geometries and loading configuration (i.e., wire loop, knife edge, blade, hook, point of loading, alignment, stressing rate) and modulus of elasticity of the evaluated materials. 34 Since the present study applied the fatigue loading using a different testing device, the results presented here could have been affected by it, in a similar way for both evaluated groups.

Comparing the loading method for shear bond strength test, the wire-loop, notched-rod, and knife-edge chisel are usually reported as the most preferred blade designs. 35 Since the knife-edge chisel transmits the force to the tested sample from a single point, a heterogeneous force transmission is observed. Despite that, the failure mode between the different application methods seems to be similar. 35 According to the literature, problems related to the validity of shear bond strength values started to arise as cohesive failures in the substrate were frequently observed with adhesives that yield improved bond strengths. 36

Therefore, as expected, not only the loading setup should affect the failure mode and force distribution, but also the evaluated materials. 33 34 35 36 37 38 39 Therefore, further fatigue bond-strength tests should be developed, assessing not only the reliability of bonded restorations but also the loading method stresses.

Considering the limitations of an *in vitro* study, such as the use of an accelerated life test and nonanatomic specimens, further investigations could be considered to analyze the influence of the cement thickness in conditions simulating anatomical specimens, as well as the behavior under different surface treatments, different ceramic materials, and varying load profiles in the adhesively cemented restorations.

## Conclusion

The resin cement thicknesses bonded to leucite ceramic did not influence the long-term interfacial shear bond strength, although thicker cement layer increased the chances of ceramic cohesive failure. Regardless the cement layer thickness, the shear bond strength lifetime decreases under fatigue.
